# *In Vivo* Functional Genomic Studies of Sterol Carrier Protein-2 Gene in the Yellow Fever Mosquito

**DOI:** 10.1371/journal.pone.0018030

**Published:** 2011-03-18

**Authors:** Rong Peng, Vilena I. Maklokova, Jayadevi H. Chandrashekhar, Que Lan

**Affiliations:** 1 College of Life Sciences, Central China Normal University, Wuhan, Hubei, China; 2 Department of Entomology, University of Wisconsin, Madison, Wisconsin, United States of America; 3 Division of Pharmaceutical Sciences, School of Pharmacy, University of Wisconsin, Madison, Wisconsin, United States of America; New Mexico State University, United States of America

## Abstract

A simple and efficient DNA delivery method to introduce extrachromosomal DNA into mosquito embryos would significantly aid functional genomic studies. The conventional method for delivery of DNA into insects is to inject the DNA directly into the embryos. Taking advantage of the unique aspects of mosquito reproductive physiology during vitellogenesis and an *in vivo* transfection reagent that mediates DNA uptake in cells via endocytosis, we have developed a new method to introduce DNA into mosquito embryos vertically via microinjection of DNA vectors in vitellogenic females without directly manipulating the embryos. Our method was able to introduce inducible gene expression vectors transiently into F0 mosquitoes to perform functional studies *in vivo* without transgenic lines. The high efficiency of expression knockdown was reproducible with more than 70% of the F0 individuals showed sufficient gene expression suppression (<30% of the controls' levels). At the cohort level, AeSCP-2 expression knockdown in early instar larvae resulted in detectable phenotypes of the expression deficiency such as high mortality, lowered fertility, and distorted sex ratio after induction of AeSCP-2 siRNA expression *in vivo*. The results further confirmed the important role of AeSCP-2 in the development and reproduction of *A. aegypti*. In this study, we proved that extrachromosaomal transient expression of an inducible gene from a DNA vector vertically delivered via vitellogenic females can be used to manipulate gene expression in F0 generation. This new method will be a simple and efficient tool for *in vivo* functional genomic studies in mosquitoes.

## Introduction

Disease vector mosquitoes (Diptera: Culicidae) are amongst many insect species detrimental to human health. The yellow fever mosquito, *Aedes aegypti*, is of specific concern because it transmits yellow fever as well as dengue hemorrhagic fever (DHF). Potential populations of 2 billion people living in tropical and sub-tropical regions are at risk for these mosquito-borne viral diseases [1]. Information from the complete mosquito genomic sequences promises to help us understand various aspects of vector mosquito biology. However, the difficulties of performing large scale functional genomic studies in vector mosquito species include the lack of highly efficient genomic tools to manipulate genes in mosquitoes as well as the lack of mutants. For example, there are 1561 orthologs that are unique to *Anopheles gambiae* and *Aedes aegypti* mosquitoes that are not shared with *Drosophila* [2]. Furthermore, *Culex quinquefasciatus* shares 10% and 2% of ortholog genes exclusively with *Ae. aegypti* and *An. gambiae*, respectively [3]. The function of those mosquito-specific genes cannot be studied in the *Drosophila melanogaster* model system. There is an urgent need to develop methods to study the function of those unique mosquito genes *in vivo*.

The method to deliver genes into mosquitoes is similar to the techniques used in traditional *Drosophila melanogaster* model systems that involve microinjection of transposon/transposase DNA vectors into the embryo [4], [5], [6]. The cumbersome nature of microinjection in mosquito embryos is compounded by the fact that mosquito eggs cannot withstand the dechronization process used for *Drosophila melanogaster* eggs [4], [5], [6]. The reported transformation rate for *Aedes aegypti* is very low (4–10%), and to generate a transgenic line in mosquitoes requires microinjection of hundreds of embryos [5], [6]. It would be an extremely labor intensive task if the function of over a thousand genes are to be studied *in vivo* in mosquitoes using the traditional DNA microinjection methods.

Female vector mosquitoes use nutrients from a bloodmeal to sustain reproductive needs. A blood meal initiates the vitellogenic process in which yolk proteins are deposited in the developing ooctyes. Vitellogenin, a protein that composes the bulk of egg yolk in mosquito eggs, is synthesized in the fat body, excreted into the hemolymph, and taken up by developing oocytes prior to formation of the egg chorion [7]. Synthesis of vitellogenin in the fat body reaches its highest levels at approximately 24 hours post-bloodmeal, whereas the uptake of yolk proteins from the hemolymph into the oocyte has a window period from 6 to 30 hours post-bloodmeal [7], [8]. Prior to vitellogenesis, the resting mosquito oocytes are sheathed by a layer of follicle cells and the resting oocytes do not uptake hemolymph proteins [9], [10]. During vitellogenesis, follicle cells surrounding the developing oocytes undergo a process termed “patency” during which the shrinkage of follicle cells forms channels between cells, allowing the oocyte direct access to hemolymph vitellogenin [9], [10], [11]. Developing oocytes import vitellogenin from the hemolymph via receptor mediated endocytosis [10], [12]. Active endocytosis in oocytes during vitellogenesis can be detected via the incorporation of labeled proteins and particles from the hemolymph [9], [10].

We hypothesized that a DNA vector may be incorporated into the oocyte when the DNA is injected into the hemolymph during vitellogenesis. Each female mosquito can produce on average 86 eggs per reproduction cycle [13]. Oogenesis of the eggs in vector mosquitoes is synchronized because vitellogenesis is triggered by the bloodmeal. Therefore, if the DNA vector is delivered via the female's hemolymph into eggs during vitellogenesis, almost all eggs would incorporate the DNA vector uniformly. We term this as a “vertical DNA vector delivery method”.

Based on the hypothesis described above, we tested whether DNA plasmids injected into the hemolymph of vitellogenic females would be taken up by developing oocytes. We also examined whether a vertically delivered DNA vector would persist in the F0 generation. The F0 larvae showed a high frequency of carrying the vertically delivered DNA vector. We have made significant advances in developing a simple and efficient DNA vector delivery method in *Aedes aegypti*. This method of DNA vector delivery will allow us to perform inducible over-expression or expression knockdown in mosquito larval and adult stages without transgenic lines.

## Results

### Delivery of inducible over-expression of DNA vector into F0

**Figure 1:**
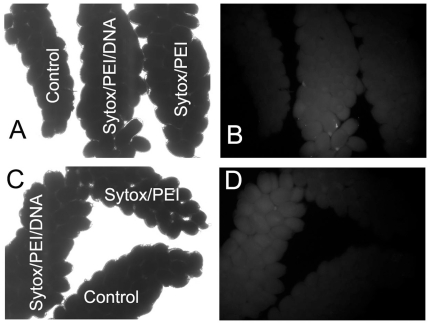
DNA uptake in developing oocytes. (**A**) Under the optical light under 40X magnification. (**B**) The same image as in panel A but under the fluorescent light. (**C**) Under the optical light under 40X magnification. (**D**) The same image as in panel C but under the fluorescent light. Females were microinjected with 0.5 µl of Sytox/PEI/DNA or Sytox/PEI complex at 18 hours post bloodmeal (PBM) or without injection (control). The ovaries were dissected out at 42 hours PBM and observed under the fluorescent microscope (Nikon DiaPhot, Nikon Inc., Melville, NY).

Linear polyethylenimine (PEI) is a cationic lipid that mediates DNA uptake in cells via binding to membrane-associated proteoglycans [14]. To visualize DNA vector uptake in the eggs, we stained the DNA with Sytox green (Invitrogen). Figure 1 shows that DNA uptake in oocytes was readily achieved at 24 hours post-bloodmeal (PBM) (Fig. 1B and D), consistent with the reports that the uptake of hemolymph molecules and yolk proteins in oocytes peaks at around 24 hour PBM [7]. Based on the visual observation under fluorescent microscope of paired ovaries for Sytox/PEI/DNA uptake, the optimal time for high efficiency of jetPEI/DNA uptake in the ovary was between 16–20 hours PBM (data not shown). The N/P ratio is a measure of the ionic balance of the complexes of jetPEI and DNA, which refers to the number of nitrogen residues of jetPEI™ per DNA phosphate. To optimize the N/P ratio for PEI-mediated DNA uptake in oocytes of vitellogenic females, we tested N/P = 7 and N/P = 10 with varied amounts of DNA vector (0.1 to 0.5 µg/female) injected into the hemocoel at 16–24 hour PBM. Significantly higher frequencies of DNA transfer occurred in F0 larvae microinjected with N/P = 10 at 0.25 µg DNA vector/female (*p* = 0.003, N = 3–9 for N/P = 7 and N/P = 10, respectively).

**Figure 2:**
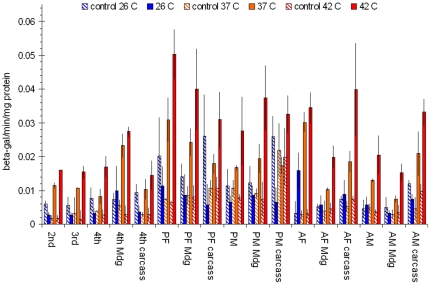
Induction of β-gal activity by heat shock in F0 from eggs of pXH70ZT/PEI microinjected vitellogenic females. Mdg =  the midgut samples; carcasses = the body wall samples; 2nd =  2^nd^ instar; 3^rd^ =  3^rd^ instar; 4^th^ =  4^th^ instar; PF =  female pupae; PM =  male pupae; AF =  adult females; AM =  adult males. Bars =  mean± S.D. (N = 3).

To determine if a non-transposable DNA vector delivered to vitellogenic females could be used to over-express a heterogeneous gene in the next generation (F0), a β-galactosidase (β-gal) reporter gene construct driven by the 194 bp hsp70 promoter (pXH70ZT) [15] was used to monitor β-gal gene expression from DNA vectors in F0. The jetPEI/pXH70ZT complex was microinjected into the hemocoel in 10–15 vitellogenic females at 24 hours PBM via the thorax. The eggs from microinjected vitellogenic females were hatched 5 days after egg deposition, and larvae (F0) were reared in water plus fish food as described [16]. At the indicated developmental stages, larvae, pupae, and adults were collected and heat shocked for 2 hours at either 37°C or 42°C. Whole organism samples were from 30 larvae of 2^nd^ and 3^rd^ instars, whereas 10 individuals were pooled from each stage of 4^th^ instars, pupae, and adults. Each tissue sample was pooled from 20–30 individuals. The β-gal activity assay was performed as described [15]. The Hsp70-β-gal construct has been shown in cell cultures to respond to 42°C heat induction [15]. In F0 individuals, heat shock treatments for 2 hours induced the expression of β-gal at all developmental stages (Fig. 2). At 37°C, the heat shock treatment induced β-gal activities in each sample and were significantly higher (p<0.05) than that of at 26°C (2- to 4-fold; Fig. 2, 26°C vs. 37°C). In most cases, heat shock at 42°C induced significantly higher levels of β-gal activities than those that were heat shocked at 37°C (Fig. 2, 42°C vs. 37°C). Heat shock at 37°C for 2 hours did not cause higher mortality than those that remained at 26°C, showing no adverse effect on the mosquitoes. Mosquitoes that underwent heat shock treatment at 42°C for two hours recovered very slowly and showed a significantly higher mortality rate than those that remained at 26°C. There was no significant increase in β-gal activities under the same heat shock conditions in samples from wild type organisms (Fig. 2 26°C control vs. 37°C or 42°C controls). The results indicate that the DNA vector injected into vitellogenic females was vertically transferred into eggs and the DNA construct persisted in F0 through larval to adult stages. Both the midgut and the carcass (the body-wall) tissues seemed to contain the vertically transferred DNA vector based on the equal responsiveness of β-gal expression to heat shock treatments (Fig. 2, Mdg and carcass). The results suggest that transient expression of an inducible gene from a DNA vector vertically delivered via vitellogenic females can be used to over-express a heterogeneous gene in F0. Using the “vertical DNA vector delivery method”, each transient expression experiment required the microinjection of 10–15 vitellogenic females which technically can be accomplished within 30 minute time frame.

### Delivery of inducible expression knockdown DNA vector into F0

To determine if the vertically delivered DNA vector can be used to induce expression knockdown of a targeted gene *in vivo*, we constructed an hsp70-AeSCP-2-siRNA expression vector. The transcription of 21 bps AeSCP-2 hairpin RNA of the coding region was under the control of the 1.1 kb *Drosophila* hsp70 promoter [15]. It is known that *Aedes aegypti* sterol carrier protein-2 (AeSCP-2) gene played an important role in the mosquito's development and reproduction, which is most likely due to AeSCP-2's function involved in cholesterol uptake [17], [18]. Results from previous studies using microinjection of dsRNA technique in larvae and adults showed that expression knockdown of AeSCP-2 gene led to higher mortality and lower fertility [17]. The jetPEI/AeSCP-2 siRNA expression vector complex was microinjected into the hemocoel of 10-15 vitellogenic females at 16–17 hours PBM via the thorax. The control females were injected with the complex of jetPEI/vector without siRNA insertion. The eggs were hatched and larvae (F0) were reared as described (Methods). At the indicated developmental stages, synchronized larvae, pupae, and adults were collected and then heat shocked at either 37°C for 24 hours or at 42°C for 3 hours. After the heat shock treatment (induction of AeSCP-2 siRNA expression), the cohorts were returned to 26°C and reared until sample collection. Pooled samples of 30 2^nd^ or 3^rd^ instar larvae or 10 4^th^ instar larvae or 30 midguts from 4^th^ instar larvae or 10 pupae or 10 adults were collected at different time points after the heat shock. Total RNAs were extracted from each sample and treated with DNaseI as described (Method). To quantify the relative levels of AeSCP-2 mRNA in different samples, we used RpL8 and Actin-1 as the internal controls for larval/pupal/adult and adult PBM samples, respectively.

**Figure 3:**
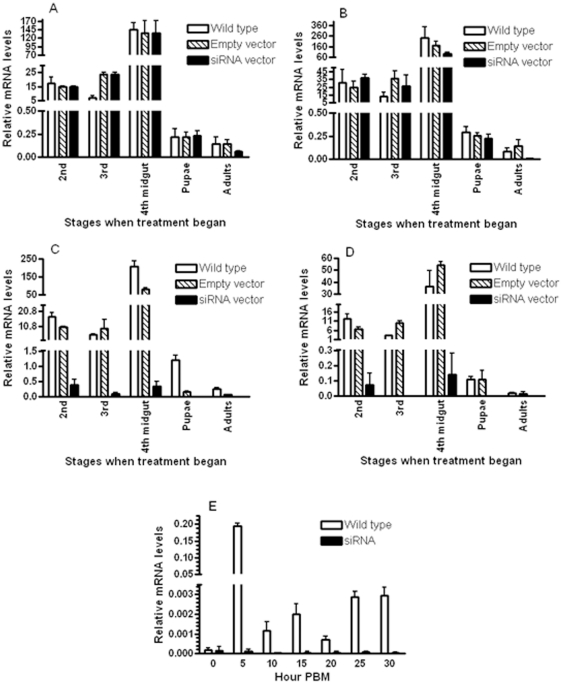
Effects of induced AeSCP-2 siRNA expression on AeSCP-2 mRNA levels *in vivo*. (**A**). At 26°C through out. (**B**). At 37°C for 24 hours. (**C**) At 42°C for 3 hours, then returned to 26°C for 24 hours. (**D**). At 42°C for 3 hours, then returned to 26°C for 48 hours. (**E**). At 42°C for 3 hours on day 1 2^nd^ instar, then returned to 26°C through the developmental stages. Bars =  Mean and standard deviation (n = 3).

Under the un-induced conditions (26°C), there were no significant differences in AeSCP-2 mRNA levels between wild type (no DNA vector), hsp70 DNA vector control, and hsp70-AeSCP-2-siRNA vector (Fig. 3A, wild type and empty vector). High levels of AeSCP-2 mRNA were detected in the 4^th^ instar midgut (Fig.3A, 4^th^ midgut), which is consistent with previous observations [19]. There were slight decreases of AeSCP-2 transcripts (1∼2-fold at most) in some AeSCP-2 siRNA vector carrying groups (Fig. 3A, siRNA vector, 4^th^ midgut and pupae). Induction of AeSCP-2 siRNA expression at 37°C for 24 hours did not significantly (Student's t-test, *p*>0.05) affect AeSCP-2 mRNA levels in larvae or pupae (Fig. 3B), indicating that the 1.1 kb hsp70 promoter might not have high transcriptional activity to generate sufficient AeSCP-2 siRNA to knockdown AeSCP-2 expression. Twenty four hours after heat shock at 42°C for 3 hours, the levels of AeSCP-2 mRNA were significantly lower (Student's t-test, *p*<0.05) at all stages examined in F0 born to females that had microinjection of the jetPEI/hsp-AeSCP-2-siRNA vector (Fig. 3C). Importantly, the high level expression of AeSCP-2 in the 4^th^ instar larval midgut was sufficiently knocked down (Student's t-test, *p*<0.05; Fig. 3C, siRNA vector, 4^th^ midgut). There were slightly lowered AeSCP-2 mRNA levels at some stages in DNA vector controls (1/2- to 7-fold) than that of in the wild type control (Student's t-test, *p*<0.05). However, the vector controls still had 15- to 230-folds higher AeSCP-2 mRNA levels (Student's t-test, *p*<0.05) than that of in the siRNA vector samples (Fig 3C, empty vector vs. siRNA vector). Forty-eight hours after the heat shock, AeSCP-2 mRNA levels in the wild type and vector controls were similar (Student's t-test, *p*>0.05; Fig. 3D, wild type vs. empty vector). However, 48 hours after induction of AeSCP-2 siRNA expression, the levels of AeSCP-2 were still significantly lower (Student's t-test, *p*<0.05) in AeSCP-2 siRNA-treated groups than that of controls (Fig. 3D).

Whether the AeSCP-2 siRNA expression induced during 2^nd^ instar would persist to the adult stage is unknown. To investigate the persistence of induced siRNA expression *in vivo*, we heat shocked 2^nd^ instar larvae at 42°C for 3 hours, and then returned the larvae to 26°C and allowed them to develop to the adult stage. We examined AeSCP-2 expression levels in adults emerged from larvae in which AeSCP-2 siRNA expression was induced at 2^nd^ instar. Four days after the adult emergence, females were given a bloodmeal, and pooled samples of 10 individuals were collected at 5 hour intervals until 30 hours PBM. Total RNAs were extracted the treated with DNaseI, and reverse transcribed into cDNAs as describe (Methods). Q-PCR analysis of AeSCP-2 mRNA levels indicated that AeSCP-2 siRNA expression induced during 2^nd^ instar had persisted to the adult stage and effectively suppressed bloodmeal-induced AeSCP-2 expression (Student's t-test, *p*<0.05; Fig. 3E, control vs. siRNA). The results were similar to reported knockdown expression by AeSCP-2 dsRNA microinjection in females [17]. Therefore, the vertically delivered inducible AeSCP-2 siRNA expression vector had functioned well in suppressing AeSCP-2 expression in through larval to adult development.

### Efficiency of delivery DNA vectors and expression knockdown in F0

To estimate the efficiency of vertically delivered DNA vector into the F0, we randomly selected individual 4^th^ instar larva from a cohort of independent batches of eggs from females microinjected with the jetPEI/hsp70-AeSCP-2-siRNA vector. A cohort of wild type larvae was used as the control group since the levels of AeSCP-2 mRNA between wild type control and vector controls were similar at 48 hours post heat shock (Fig. 3D). The wild type control group was heat shocked as that of the F0 larvae born to jetPEI/siRNA vector microinjected females. The cohort of F0 2^nd^ instar larvae were heat shock at 42°C for 3 hours, and then returned to 26°C. At 48 hours after the induction of AeSCP-2 siRNA expression, 10 larvae were selected randomly and the levels of AeSCP-2 mRNA in each larva were determined via qPCR analysis. On average, 48 hours of AeSCP-2 siRNA-treatment resulted in significantly lowered levels of AeSCP-2 mRNA in F0 than that of the control group (Student's t-test, p<0.05; Table 1, Batch#1, mean/S.D.). At the individual level, 90% of AeSCP-2 siRNA-treated F0 larvae had lower AeSCP-2 mRNA levels than the average of the controls. Overall, 70% and 50% of AeSCP-2 siRNA-treated larvae had less than 30% and 15% levels of AeSCP-2 transcripts than that of the average in the control, respectively (Table 1, Batch#1). In a repeated experiment, 15 larvae were selected randomly at 72 hours after the induction of AeSCP-2 siRNA expression. In the control group of Batch#2, 2 individual larvae were newly molted 4^th^ instar and had significantly lower AeSCP-2 mRNA levels than the rest of day1 4^th^ instar larvae in the control group (Student's test, *p*<0.05; Table 1, Batch#2 larvae marked with** vs. the rest of the controls). The results were consistent with the observation that AeSCP-2 expression is significantly lower in newly molted wild type 4^th^ instar larvae [20]. On average, at 72 hours after the induction of siRNA expression F0 larvae had significantly lower levels of AeSCP-2 mRNA than that of the control (Students' t-test, *p*<0.05; Table 1, Batch#2, mean/S.D.). At the individual level, 72 hours after the induction of AeSCP-2 siRNA expression 73% and 47% of siRNA-treated larvae had lower than 30% and 15% levels of AeSCP-2 transcripts compared to that of the average of the control, respectively (Table 1, Batch#2, siRNA induced). Interestingly, developmental delays were observed in AeSCP-2 siRNA-treated larvae in which larvae in AeSCP-2 siRNA expressing groups were at least 12 hours developmentally behind the control groups (Table 1, “Stages” control vs. siRNA induced). When the comparable developmental stages were compared, AeSCP-2 siRNA-treated larvae had significantly lower AeSCP-2 mRNA levels than that of control in the late 3^rd^ instar (Student's t-test, *p*<0.05; Table 1, Batch#1 control vs. Batch#2 siRNA induced). The results showed that the vertically delivered inducible AeSCP-2 siRNA expression vector efficiently (in >70% F0 individuals) knocked down the target gene expression in F0 larvae. The results also indicate that the strength of siRNA expression knockdown was similar at 48 hours as at 72 hours after the induction.

**Table 1:** Efficiency of vertical DNA delivery to F0 individual 4^th^ instar larva (relative AeSCP-2 mRNA levels vs. rpL8).

	Batch#1 (larvae/48 hr PSI)*		Batch#2 (larvae/72 hr PSI)*	
	control	siRNA induced	control	siRNA induced
1	8.77	5.79	4.81	1.52
2	9.19	2.30	5.79	0.55
3	8.00	4.59	6.20	1.45
4	7.13	2.19	9.62	2.83
5	7.29	0.98	4.29	0.31
6	8.77	4.49	7.82	2.05
7	9.19	0.68	7.82	0.17
8	5.79	0.17	5.79	0.10
9	8.77	0.52	5.28	0.03
10	5.28	0.35	1.18**	1.05
11	–	–	7.46	2.41
12	–	–	3.32	0.95
13	–	–	3.48	0.59
14	–	–	2.96	2.30
15	–	–	1.91**	0.19
stages§	Late 3^rd^	Early 3^rd^	Early 4^th^	Late 3^rd^
mean	7.82	2.21	5.18	1.10
S.D.	1.41	2.06	2.30	0.91
t-test		*p* = 0.000001		*p* = 0.000001

*PSI = post siRNA induction: synchronized day1 2^nd^ instar larvae were heat shocked at 42°C for 3 hour and returned to 26°C for the duration before sample collection. Control =  wild type individuals experienced identical treatment as the siRNA induced groups.

**Newly molt 4^th^ instar larva.

§ The development stage when samples were collected.

### Effect of AeSCP-2 siRNA induction on development and reproduction

Earlier studies have shown that expression knockdown of AeSCP-2 in 4^th^ instar larvae leads to high mortality in early adults and lowered fertility [17]. In order to verify the effects of AeSCP-2 siRNA expression on the development progression and fertility in the F0 generation, AeSCP-2 siRNA expression was induced in day1 2^nd^ instar larvae by heat shock at 42°C for 3 hours, and then returned to 26°C. The control group was the wild type larvae treated under the same conditions as the F0 transiently inherited the hsp-AeSCP-2 siRNA vector. After the heat shock, 30 synchronized larvae (based on the time of molting) from each group were placed into a clean 200 ml beaker with 150 ml distilled water and were fed the same amount of food. In a separate experiment, a cohort of synchronized day1 4^th^ instar larvae were heat shocked at 42°C for 3 hours, and then returned to 26°C. Developmental progress (stages) and mortality were observed daily.

Heat shock at 42°C for 3 hours on day 1 of the 2^nd^ instar resulted in 23.5±6.0% (mean±S.D.) larval mortality through the larval stages in the control group, whereas the AeSCP-2 siRNA-treated group had 37.5±5.0% (mean±S.D.) larval mortality (Table 2, started at 2^nd^ instar, Total mortality/larvae). The mortality differences between the control and the AeSCP-2 RNAi-treated groups were significant in larvae (*df* = 14, *t* = 2.67, *p*<0.05). Overall, mortalities in AeSCP-2 siRNA-treated groups starting in the 2^nd^ instar were significantly higher than that of the controls (*F*_1,18_ = 13.83, *p* = 0.0016). Heat shock at 42°C for 3 hours on day 1 4^th^ instar led to 10% larval mortality in the control group, whereas the AeSCP-2 siRNA-treated group had 30% larval mortality (Table 2, started at 4^th^ instar, Total mortality/Larvae). Similarly, AeSCP-2 siRNA-treatment starting in 4^th^ instar resulted in higher pupal/early adult mortality than that of the control (Table 2, started at 4^th^ instar, Total mortality/P+ early A). The higher mortality rate in AeSCP-2 siRNA treated larvae is consistent with observations of higher early adult mortalities from AeSCP-2 dsRNA microinjection in day 1 4^th^ instar larvae [17].

**Table 2:** Effects of AeSCP-2 siRNA expression on development.

	batch	3^rd^ day			7^th^ day			11^th^ day				Total mortality		
		3^rd^	4^th^	dead	4^th^	pupa	dead	4^th^	pupa	adult	dead	larvae	P+ early A	total
Control, started at 2^nd^ instar (N = 30/batch)	#1	7	20	3	3	21	3	0	0	20	4	20% (6)*	17% (4)	27%
	#2	5	22	3	8	17	3	0	2	19	7	30% (9)	5% (1)	35%
	#3	8	19	3	7	20	2	0	0	22	4	27% (8)	9% (2)	36%
	#4	5	22	3	3	23	1	0	0	23	3	17% (5)	8% (2)	25%
Induced siRNA at 2^nd^ instar (N = 30/batch)	#1	26	1	3	10	13	4	3	5	11	5	30% (9)	29% (6)	59%
	#2	23	2	5	17	2	6	0	8	10	1	40% (12)	6% (1)	46%
	#3	22	3	5	17	3	5	0	6	12	2	40% (12)	6% (1)	46%
	#4	22	2	6	18	2	4	2	5	11	2	40% (12)	6% (0)	40%


	3^rd^ day				7^th^ day			8^th^ day			Total mortality		
	4^th^	pupa	adult	dead	pupa	adult	dead	pupa	adult	dead	larvae	P+ early A	total
Control, started at 4^th^ instar (N = 30)	0	20	7	3	0	24	3	0	22	2	10% (3)	17% (5)	27%
Induced siRNA at 4^th^ instar (N = 30)	3	21	0	6	3	15	6	0	16	2	30% (9)	24% (5)	54%

*Number in () indicates the actual number of fatality in the cohort.

AeSCP-2 siRNA-treated larvae developed at a slower pace than that of the control. Three days after heat induced AeSCP-2 siRNA expression in 2^nd^ instar, most of the surviving larvae in the control group had molted into 4^th^ instar, whereas most of the AeSCP-2 siRNA-treated larvae remained as 3^rd^ instar larvae (Table 2. started at 2^nd^ instar, 3^rd^ day). Some larvae that experienced AeSCP-2 siRNA expression induced on day1 2^nd^ instar were still in the larval stage when all surviving individuals in the control groups had emerged as adults (Table 2. started at 2^nd^ instar, 11^th^ day). Those 11^th^ day larvae eventually died as pupae (Table 2, started at 2^nd^ instar, Total mortality/P+ early adult). The delay of developmental progression as a cohort to reach certain developmental stages was significant between AeSCP-2 siRNA-treated larvae starting in day1 2^nd^ instar and the control (*F*_1,18_ = 153.4, *p*<0.0001). Induction of AeSCP-2 siRNA expression on day1 4^th^ instar did not significantly delay the pupation or adult emerge in the siRNA-treated group (Table 2. started at 4^th^ instar, 3^rd^ day and 7^th^ day), which is consistent with the observation in AeSCP-2 dsRNA microinjected day 1 4^th^ instar larvae [17]. Therefore, the accumulative effect of AeSCP-2 siRNA expression on the development of larvae, pupation, and adult emerge was only observed in the group that experienced AeSCP-2 siRNA expression induction at an early larval stage. Surprisingly, the sex ratio in adults from AeSCP-2 siRNA-treated larvae was female biased. Adults emerged from wild type larvae heat shocked on day1 2^nd^ or 4^th^ instar were 0.84∶1 and 0.83∶1 of male:female, respectively (Table 3, controls male vs. female). Adults emerged from AeSCP-2 siRNA-treated larvae heat shock on day1 2^nd^ or 4^th^ instar were 0.49∶1 and 0.45∶1 of male:female, respectively (Table 3, siRNAs male vs. female). Differences in sex ratio between adult emerged from AeSCP-2 siRNA-treated larvae and from the controls were significant (Student's t-test, *p*<0.05). The results suggest that AeSCP-2 expression in larvae may be much more critical for the survival of developing males than that of for the females.

**Table 3:** Effects of AeSCP-2 siRNA expression on reproduction.

From 30 larvae/batch to 5 days after adult emergence	batch	male	Female	Fed BM	eggs	Eggs/Female	Hatched	% hatch	Fertility*
Control, started at 2^nd^ instar	#1	9	10	9	642	71	497	77.41%	55
	#2	10	10	9	672	67	537	79.91%	54
	#3	9	12	11	772	64	627	81.22%	52
	#4	10	13	13	827	64	665	80.41%	52
Induced siRNA at 2^nd^ instar	#1	3	14	11	526	48	332	63.12%	30
	#2	6	11	9	437	40	223	51.03%	20
	#3	5	12	10	495	41	257	51.92%	21
	#4	6	12	10	507	42	261	51.48%	22
Control, started at 4^th^ instar		10	12	11	809	74	597	73.79%	55
Induced siRNA at 4^th^ instar		5	11	9	646	72	337	52.17%	38

*Fertility =  viable F1 offspring/female (larvae were countered during 2^nd^-3^rd^ instar).

Adults from each treatment group were caged together and mated within the group. Females from the control and AeSCP-2 siRNA-treated groups took the bloodmeal at the similar rates (Table 2, Fed Blood), indicating that AeSCP-2 expression knockdown did not affect the bloodmeal feeding behavior. However, females emerged from larvae that experienced AeSCP-2 expression knockdown starting on day1 2^nd^ instar had significantly lower fecundity than that of the controls (Table 3. started siRNA at 2^nd^ instar, eggs/female, *df* = -23.75, *t* = 8.99, *p*<0.001). There was no differences in fecundity between females from AeSCP-2 siRNA-treatment starting in day1 4^th^ instar and the control (Table 3. started siRNA at 4^th^ instar, eggs/female), which is consistent with the observation in dsRNA injected early adults [17]. Hatching rates were lower in eggs from females of AeSCP-2 siRNA-treated larvae than that of controls. The overall female reproductivity (fecundity, % egg hatching rate, fertility) of adult females from AeSCP-2 siRNA-treated larvae was significantly lower than that of controls (*F*_1,18_ = 289.6, *p*<0.0001). The results are consistent with the observation of lowered fertility in dsRNA treated adult females [17].

## Discussion

One of the major obstacles in a large scale functional genomic study with vector mosquitoes is the lack of a simple and efficient method to manipulate gene expression *in vivo*. Microinjection techniques have shown improvement regarding embryo hatching and survival rate using the traditional “horizontal DNA vector delivery method”, producing a 10% transformation frequency [6]. However, due to the low hatching (40–50%) and survival rate (30–40%) of the larvae from microinjected embryos [6], large-scale transformations of thousands of genes in mosquitoes is still likely an impossible task to undertake using traditional methods. Microinjection of dsRNA have been successfully used in 4^th^ instar larvae and adults [17], [18], however, inducible gene expression knockdown in early instar stages is not possible with microinjection of dsRNA for functional genomic studies. Extrachromosmal expression of heterogeneous genes *in vivo* has been described in *C. elegans* [21], [22], *B. mori* [23], and *A. aegypti* [24]. In fact, the only technique to deliver DNA vectors into *C. elegans* embryos is via direct microinjection of DNA into the gonads [25]. In *A. aegypti* adults, transient *in vivo* expression of microinjected heterogeneous gene has been described with high variability and low DNA transfection efficiency in the ovary [24]. In all of those described *in vivo* DNA transfection methods in arthropods, only purified DNA plasmids are used [21], [22], [23], [24]. We sought to improve the techniques for *in vivo* transient transfection by changing the DNA vector delivery method.

During vitellogenesis in *A. aegypti*, developing oocytes take up large amount of yolk proteins from the hemolymph between 6–30 hours post-bloodmeal [7], [8] and in the process importing molecules as large as 500 kDa from the hemolymph via endocytosis [9]. The oocyte is lined with mucopolysaccharide material, which leads to the selective uptake of molecules via endocytosis [9]. Linear polyethylenimine (PEI) is a cationic lipid that mediates endocytotic DNA uptake in cells via binding to membrane-associated proteoglycans [14]. We reasoned that an *in vivo* transfection reagent such as PEI (*In vivo* jetPEI^TM^, Polyplus-transfection Inc., NY, USA) might enhance uptake of DNA vectors into developing oocytes, which would deliver the DNA vector into the embryos of the next generation, the F0. We term this technique “the vertical DNA vector delivery method”.

Injection of the complex of an *in vivo* transfection reagent such as jetPEI^TM^ and a plasmid DNA vector into vitellogenic females (16–24 hours PBM) resulted in high frequencies of transfected F0 individuals judging from the frequency of sufficient siRNA mediated expression knockdown of a targeted gene in the F0 individuals (Table 1, induced siRNA vs. control). The technical improvement of the jetPEI/DNA complex over the naked DNA vector microinjection [24] is the higher efficiency of plasmid DNA uptake in the ovary (Fig. 1B and D). In a typical trail, injection of 10 vitellogenic females 16–24 hours PBM would produce about 400 eggs with about a 70–80% hatching rate, which would give rise to approximately 300 transiently transfected F0 larvae. A cohort of 300 transiently transfected F0 is sufficient for a functional genomic experiment, and the turn-around time for repeated experiments was less than 3 weeks. Furthermore, with more than 70% of the F0 individuals carrying sufficient amount of heterogeneous DNA vector (Table 1) to reliably induce over-expression or expression knockdown of targeted gene *in vivo* (Fig. 2 and 3), controlled manipulations of a gene expression *in vivo* in all development and reproductive stages can be performed (Table 3). Although the vertically delivered DNA vector was not 100% efficient in the F0 (Table 1), at the cohort level, the vertically delivered DNA vectors could be used to study genomic function of a targeted gene *in vivo* (Table 2 and 3) without creating transgenic lines. The new transient transfection method for F0 offers an inducible way for gene expression knockdown in all developmental stages (Fig. 3), which is unachievable via dsRNA microinjection technique.

There is a possibility that 42°C heat shock for 3 hours may have additional effects on the siRNA treatment, which might mediate some of the observed biological effects (Table 2 and 3). However, the heat shock-induced AeSCP-2 RNAi has similar biological effects on larvae and adults compared to AeSCP-2 dsRNA microinject [17] when comparable stages in both studies are considered. Therefore, the biological effect of AeSCP-2 siRNA in the 2^nd^ instar larvae is likely due to the suppression of AeSCP-2 gene expression. In *Manduca* GV1 cells, the 194 bp hsp70 promoter (the short) is not active at 37°C, whereas the 1.1 kb hsp70 promoter (the long) induced targeted gene expression at 37°C [15]. Both the short and long hsp70 promoters are active in *Manduca* GV1 transfected cells at 42°C [15]. We constructed the AeSCP-2 siRNA expression vector driven by the long hsp70 promoter, expecting induction of the siRNA expression at 37°C. Both the short (194 bp) and long (1.1 kb) *Drosophila* hsp70 promoter induced expression of target gene expression after heat shock at 42°C (Fig. 2 and 3). However, it is noticed that heat shock induced promoter activities of the *Drosophila* 194 bp and the 1.1 kb hsp70 promoter were different at 37°C in *A. aegypti*. Two hours heat shock at 37°C was sufficient in the induction of β-gal gene (the reporter gene) expression driven by the 194 bp hsp70 promoter (Student's t-test, *p*<0.05; Fig. 2, 37°C vs. 26°C). On the other hand, 24 hour hours at 37°C did not induce sufficient siRNA (the reporter gene) expression driven by the 1.1 kb hsp70 promoter to knockdown the AeSCP-2 expression (Fig. 3B, siRNA vector). The results are opposite what is reported in *Manduca sexta* transfected GV1 cells [15]. The difference in heat-shock temperature sensitivity of the *Drosophila* hsp70 short (194 bps) and long (1.1 kb) promoter between *Manduca* GV1 cells and *A. aegypti* may be due to the *in vivo* factors in the two different species. Heat shock at 42°C for 3 hours resulted in high larval mortality rate, especially if the heat shock was carried out at the early larval stage (Table 2, controls, Total mortality/larvae, started at 2^nd^ instar vs. 4^th^ instar), whereas at 37°C we did not observe significant mortalities in either wild type or F0. The *Drosophila* 194 bp hsp70 promoter should be investigated for its activity to drive siRNA expression at 37°C in *A. aegypti*.

The fecundity (eggs/F) of *A. aegypti* females is not influenced by fertilisation and females lay unfertilized eggs after a bloodmeal has been reported [13]. However, the quantity of nutrient uptake/larva during larval development profoundly affects fecundity in *A. aegypti* [26]. During the first gonotrophic cycle, *A. aegypti* females mobilize 75% of larval stored lipids and deposit about 35% of larval stored lipids into the first batch of eggs [27]. On the other hand, females during the first gonotrophic cycle deposit slightly over 60% of bloodmeal-derived fatty acid and cholesterol into eggs [18]. Therefore, lipids absorbed during larval growth are critical for the reproduction during the first gonotrophic cycle. Induction of AeSCP-2 siRNA expression in early instar resulted in lower fecundity than that of AeSCP-2 siRNA expression induced in late instar (Table 3, eggs/F, induced siRNA at 2^nd^ instar vs. at 4^th^ instar). The lowered fecundity in females from larvae that experienced AeSCP-2 expression knockdown for a longer period during larval growth might have accumulatively much lower levels of cholesterol storage because AeSCP-2 plays an important role in cholesterol uptake in larvae [17]. The long persistence of the induced AeSCP-2 siRNA expression through larval, pupal, and adult stages (Fig. 2E) might have also affected cholesterol uptake from the bloodmeal. Previous study shows that AeSCP-2 dsRNA knockdown of AeSCP-2 expression in adult females results in significantly reduced cholesterol uptake from a bloodmeal [18]. However, there was no decrease of fecundity in females from larvae induced AeSCP-2 siRNA expression in late instar (Table 3, eggs/F, induced siRNA at 4^th^ vs. controls). Therefore, the fecundity in the first gonotrophic cycle was only affected by the duration of AeSCP-2 expression knockdown in larval stages. Results from AeSCP-2 expression knockdown in early larval stages suggest that a larval stored cholesterol reverse might be one of the critical factors that influence fecundity in the first gonotrophic cycle. This is the first report of the function of AeSCP-2 in fecundity in *A. aegypti*. Whether larval stored cholesterol reserve is important for the subsequent reproductive cycles needs further investigation.

*A. aegypti* adult sex ratio in the wild populations is close to 1∶1 of male: female [28], which is similar to the control groups in this study (Table 3, controls, male/female). However, adults that survived the AeSCP-2 siRNA expression in larval and pupal stages had a female biased sex ratio (Table 3, induced siRNA at 2^nd^ and 4^th^ instar, male/female). There was a higher larval mortality rate in AeSCP-2 siRNA-treated groups (Table 2, Total mortality), which led to slightly lowered larval densities than that of the control groups. However, a previous report shows that the sex ratio in the *A. aegypti* Rockefeller strain is not affected by larval density or rearing temperature [29]. Therefore, the distortion of sex ratio in AeSCP-2 siRNA-treated groups was likely due to the suppressed levels of AeSCP-2 expression in larval and pupal stages. The results suggest that the survival of male progeny may be affected by the function of AeSCP-2. The lipid content in pupae represents larval lipid storage levels since pupae are in a non-feeding stage. The lipid reserve in *A. aegypti* pupae is sex dimorphic in which the lipid reverse is more 33% higher in males than that of in females, even though the body weight of males is 44% lower than that of females [30]. *A. aegypti* larval lipids reserve includes cholesterol [31] and deficiency in dietary cholesterol has lethal effects on *A. aegypti* larvae [30]. We have shown that AeSCP-2 selectively mediates cellular cholesterol uptake [18], [32]. It is possible that developing male larvae require the storage of much higher amount of cholesterol than that of females and AeSCP-2 mediated cholesterol uptake from the dietary sources becomes a much more critical factor for the survival of males. This is the first report on the functional importance of AeSCP-2 for the development of male progeny in mosquitoes. We did not check the sex ratio in pupae during the experiment and only the sex of the surviving adults was recorded. Whether the sex ratio distortion in AeSCP-2 siRNA-treated larvae happens by the pupal stage needs further investigation. Whether AeSCP-2 expression knockdown results in much lower cholesterol storage in male pupae also needs further study.

In summary, we have developed a simple and efficient DNA delivery method to introduce expression vectors into embryos and F0 progenies. The efficiency of transient *in vivo* DNA transfection in F0 individuals was greater than 70% (Table 1), which allows observation of phenotypic changes in a cohort (Table 2 and 3) without creating transgenic lines. Inducible *in vivo* expression of over-expression and expression knockdown was detected at high efficiency in F0 progenies (Fig. 2 and 3). Promoters that can drive tissue-specific expression of a reporter gene were also tested using the vertical DNA delivery method, the results showed that tissue-specific manipulation of gene expression in F0 is also possible using this method (Rong Peng and Que Lan, in preparation). An added advantage of transient *in vivo* DNA transfection is that the DNA expression vector is extrachromosomal, therefore, the expression of a targeted gene on the DNA vector would not be affected by the genetic background of genomic DNA. The method was developed in *A. aegypti*, however, preliminary tests in *Anopheles gambiae* showed that the method could be optimized for transient *in vivo* transfection in F0 *Anopheles* (Susan Paskewitz, personal communication). Vector mosquitoes have a very similar gonotrophic cycle that is triggered by a bloodmeal, it is highly likely that this vertical DNA delivery method could be applied to other vector mosquito species in which functional genomic studies can be performed without the time consuming process of creating transgenic lines.

## Methods

### Chemicals and reagents

Chemicals and reagents were purchased from Sigma (St. Louis, MO), Fisher Scientific (Pittsburgh, PA) and ICN (Costa Mesa, CA) if their origins are not mentioned in the text. Enzymes for manipulating DNA during cloning processes were purchased from New England Biolabs (Ipswich, MA) or Promega (Madison, WI).

### Mosquitoes

*Aedes aegypti*, the yellow fever mosquito, is from an inbred laboratory strain (Rockefeller) that was maintained at 26°C in 16 h day light/8 h night cycle in 70-80% humidity. Female adults were blood fed with defibrinated rabbit blood (Hemostat Laboratories, Dixon, CA) using glass a feeder and circulation of heated 37°C water.

### Plasmids

The pXZT70 (β-gal) construct has been previously described [15]. The 160 bp SV40 polyadenylation signal or SV40 poly(A) sequence was removed from the pIE1*^hr^* vector [33]. The pIE1*^hr^* plasmid was first cut with *Eco*RI, filled the 5′ overhang with Klenow^exo-^ (New England Biolabs), and then cut with *Sal*I, the 160 bp SV40 poly(A) DNA fragment (*Sal*I/*Eco*RI-blunt) was gel purified using the gel purification kit (Qiagen, Chatsworth, CA, USA). The pBS-hsp70 plasmid [15] was cut with *Apa*I, the 3′ overhang was repaired using Klenow (New England Biolabs), and then ethanol-precipitated. The blunt ended *Apa*I-linearized pBS-hsp70 plasmid was cut with *Sal*I and ligated to the gel purified SV40 poly(A) DNA fragment. The insertion of SV40 poly(A) into the blunt-ended *Apa*I site led to the restoration of the *Eco*RI site at the 3′ of the SV40 poly(A) in the pBS-hsp70-SV40 poly(A) plasmid. The pBS-hsp70-SV40 ploy(A) plasmid has a multiple cloning site between the hsp70 promoter and the SV40 poly(A). Duplex DNA oligo of sense and antisense nucleotides for the small hairpin RNA targeting AeSCP-2 coding region (Table 4) was synthesized (IDT, Coralville, IA), The DNA oligo was cloned into pBlunt zero vector (Invitrogen, Carlsbad, California). The cloned AeSCP-2 siRNA was cut out of the pBlunt plasmid using *Pst*I and *Sal*I to generate a new *Pst*I site at the 5′end for directional cloning into the pBS-hsp70-SV40 poly(A) expression vector. Restriction enzyme digestion and DNA sequencing confirmed the AeSCP-2 siRNA insertion.

**Table 4:** Primers and oligo sequences for qPCR and AeSCP-2 siRNA.*

Gene	
Rpl8	F: 5′-TACCTGAAGGGAACCGTCAAGCAA-3′
	R: 5′-ACAATGGTACCTTCGGGCATCAGA-3′
Actin-1	F: 5′-CCCTGAAGTACCCCAATGAGC-3′
	R: 5′-CCATGTCATCCCAGTTGGTG-3′
AeSCP-2	F: 5′- GCTGGTCGAGTCCGACGATGC-3′
	R: 5′- CAGGGCACCGGTTCCGATGG-3′
siRNA	Sense: 5′-ggaattcGTCAAGCTGGTCGAGTCCGACgggcccGTCGGACTCGACCAGCTTGaagcttggg-3′
	Antisense: 5′-cccaagcttCAAGCTGGTCGAGTCCGACgggcccGTCGGACTCGACCAGCTTGACgaattcc-3′

*The capital letters represent the sequences of targeted genes; lower case letters represent added DNA sequences for directional cloning or diagnostic restriction digest (underlined lower case letters). AeSCP-2: AY190283; AeRpL8: M99055; AeAct-1: U20287.

All of the plasmids were purified using the EndoFree plasmid Maxi Kit (Qiagen), then filtered through 0.22 µm MCE Syringe Filter (Fisher brand, cat #09-719A). The quantity of the DNA was determined using UV OD_260_ absorption on NanoDrop (NonoDrop™1000, NanoDrop Products, Wilmington, DE, USA). It was noticed that to achieve consistent results for DNA transfer from vitellogenic females to F0 larvae, an accurate DNA concentration was critical. It was noticed that EndoFree preps resulted a lower mortality rate in jetPEI/DNA injected females than that of plasmid maxi preps.

### Microinjections

Five to seven day-old mated adult females in a rearing cage were fed defibrillated rabbit blood and then microinjected using a stereo Microscope and Tilting base micromanipulator (Applied Scientific Instrumentation). The glass capillary for microinjection was made using 50 µl glass micropipets (VWR International, West Chester, PA, USA) with a Puller at heater level 40.3 for No. 1 and at heater level 48.0 for No. 2 Heater (PC-10, Narishige, Tokyo Japan). The *In vivo* jetPEI transfection reagent (PolyPlus Battery Company, Berkeley, CA, USA) was used at N/P ratio 7 and 10. The N/P ratio is a measure of the ionic balance of the complexes of PEI and DNA, which refers to the number of nitrogen residues of jetPEI™ per DNA phosphate. A total of 20 µg of plasmid DNA was diluted into 10 µl ddH_2_O and a 10 µl of 10% glucose solution was added to final 5% glucose concentration. DNA concentration was measured using NanoDrop ND-1000 before each microinjection to obtain the most accurate quantity of soluble plasmid DNA. Separately, 2.8 µl and 4 µl of *In vivo* jetPEI reagent for N/P = 7 and N/P = 10, respectively, was diluted in 10 µl ddH_2_O and a 10 µl of 10% glucose solution was added to final 5% glucose. The 20 µl of *In vivo* jetPEI solution was added into the 20 µl of DNA plasmid solution (1 µg DNA plasmid/µl) and mixed at once. The jetPEI/plasmid DNA mixture was incubated at room temperature for 15 minutes, and stored on ice during the injection process.

The mosquitoes were injected at 5–6 h, 16–18 h, 23–24 h, and 30–31 h PBM in repeated experiments. The degree of DNA/Sytox uptake in oocytes (fluorescent intensity) were observed. If a pair of ovaries had uniformed intense fluorescence compared to the controls (Fig. 1B and D, Sytox/PEI/DNA vs. Sytox/PEI or Sytox/PEI/DNA vs. control), it was score as “positive”. If the ovaries only had spotted or very weak fluorescence, it was score as “negative”. The number of mosquitoes had scored “positive” was noticed for each time points (5–5 h, 16–18 h, 23–24 h, and 30–31 h PBM). The optimal time would have most mosquitoes scored positive. For each experiment, we usually injected about 10 females. In various experiments, a minimum of 0.025 µl and a maximum of 0.5 µl jectPEI/DNA construct mix (0.025 to 0. µg DAN/female) were microinjected intrathoracially into the hemocoel of *A. aegypti* females at time points of 3, 16, 18, 22, 24, and 30 hr post the bloodmeal (PBM). The blood-fed mosquitoes were anesthetized briefly at 4°C in a container and the anesthetized mosquitoes were maintained on a chill table no more than 20 minutes prior to microinjection. Each experiment was repeated several times.

It is important that the jetPEI/DNA complex was injected into the hemocoel, not into the midgut lumen. It is suggested that an operator to be trained first in the micro-injection technique using 0.4% Trypan blue in PBS saline solution. If the Trypan blue staining in pericardial cells were vividly visible at 24 hour post micro-injection, the dye solution was corrected injected into the hemocoel. The trained operator should also be able to detect the slight blue colored ovaries from females injected with 0.4% Trypan blue, which was inspected under an optical dissecting microscope. Consistent high frequency of DNA transfer into oocytes can be obtained after an operator was technically able to achieve ≥90% success rate in delivery Trypan blue solution into the hemocoel of vitellogenic females.

### Real-time quantitative RT-PCR

The mosquito specimens from each experiment were randomly collected and placed in 1.5 ml Eppendorf tubes with Trizol reagent (Invitrogen). Larval and pupal specimens were washed in DEPC-H_2_O and excess H_2_O was blotted off prior to be placed into the Trizol reagent. Pooled samples were taken: 30 2^nd^ instar larvae/sample; 10 3^rd^ or 4^th^ instar larvae/sample; 10 pupae or adults/sample; 30 tissues/sample. The total RNA was extracted from each sample using the manufacturer's protocol and treated twice at 37°C for 30 minutes with Turbo DNA-*free* Kit (Applied Biosystems/Ambion, Austin, TX), to remove DNA contamination from the RNA preparation. RNA concentration was measured after the treatment using NanoDrop Spectrophotometer, and 0.5 µg of total RNA was used for reverse transcription reactions to generate single-stranded cDNA, using High-Capacity cDNA Archive Kit (Applied Biosystems, Austin TX).

Quantitative PCR (qPCR) was performed using the iQ™ SYBR® Green Supermix (Bio-Rad Laboratories, Hercules, CA). The PCR reaction solution contained 1 µl of cDNA from the RT reaction (equivalent to 25 ng RNA) with the following conditions: 94°C for 3 min, 40 cycles at 94°C for 10 s, 54°C for 30 s, and a final extension at 72°C for 2 min. Primers for PCR were listed in Table 4. Critical qPCR parameters of the internal controls are described (Table 4 and Table S1). The mRNA levels of AeSCP-2 in RT-qPCT analysis were compared to rpL8 or Actin-1 mRNA levels to obtain the “Relative levels”.

### Statistical analysis

Data were analyzed with two-way ANOVA (GLM procedure) to determine if several components of the biological parameter in the control groups and treated groups differed significantly using the GraphPad PRISM software version 4.0 (GraphPad). Student's t-test was used in cases where a pair of treatments was compared to determine the significance of the differences [34].

## Supporting Information

Table S1 **Q-PCR parameters of the two internal control genes.**
(DOC)
