# Supplementary material for: In Vivo Functional Genomic Studies of Sterol Carrier Protein-2 Gene in the Yellow Fever Mosquito
Source: PLoS One. 2011 Mar 18;6(3):e18030. doi: 10.1371/journal.pone.0018030 (PMC3060925; doi:10.1371/journal.pone.0018030)
Supplement: Table S1 — Q-PCR parameters of the two internal control genes. (DOC) [file pone.0018030.s001.doc]

**Table S1**. Q-PCR parameters of the two internal control genes

|  | Ct values | | | | | | | | | | | Amplicon | Efficiency |
| --- | --- | --- | --- | --- | --- | --- | --- | --- | --- | --- | --- | --- | --- |
|  | L (n=30) | | | P (n=15) | | | A (n=15) | | | A-PBM (n=35) | |  |  |
|  | W | EV | siRNA | W | EV | siRNA | W | EV | siRNA | W | siRNA |  |  |
| Rpl8 | 23.2 (±0.81) | 22.8  (±0.64) | 22.9  (±0.61) | 22.9  (±0.65) | 22.7  (±0.53) | 22.7  (±0.61) | 22.8  (±0.55) | 22.8  (±0.75) | 22.7  (±0.56) |  |  | 221 | 99.3% |
| Aeact-1 |  | | | | | | | | | 21.4  (±0.13) | 21.4  (±0.12) | 51 | 99.7% |

W= wild type (no DNA vector); EV= empty vector; siRNA= AeSCP-2 siRNA expression vector; L= larvae; P= pupae; A= adults; A-PMB= adults post bloodmeal.
